# Mining candidate gene for rice aluminum tolerance through genome wide association study and transcriptomic analysis

**DOI:** 10.1186/s12870-019-2036-z

**Published:** 2019-11-12

**Authors:** Peng Zhang, Kaizhen Zhong, Zhengzheng Zhong, Hanhua Tong

**Affiliations:** State Key Laboratory of Rice Biology, China National Rice Research Institute, Hangzhou, 310006 China

**Keywords:** Rice aluminum tolerance, Candidate gene, Genome wide association study, Transcriptomic analysis

## Abstract

**Background:**

The genetic mechanism of aluminum (Al) tolerance in rice is great complicated. Uncovering genetic mechanism of Al tolerance in rice is the premise for Al tolerance improvement. Mining elite genes within rice landrace is of importance for improvement of Al tolerance in rice.

**Results:**

Genome-wide association study (GWAS) performed in EMMAX for rice Al tolerance was carried out using 150 varieties of Ting’s core collection constructed from 2262 Ting’s collections with more than 3.8 million SNPs. Within Ting’s core collection of clear population structure and kinship relatedness as well as high rate of linkage disequilibrium (LD) decay, 17 genes relating to rice Al tolerance including cloned genes like *NRAT1, ART1* and *STAR1* were identified in this study. Moreover, 13 new candidate regions with high LD and 69 new candidate genes were detected. Furthermore, 20 of 69 new candidate genes were detected with significant difference between Al treatment and without Al toxicity by transcriptome sequencing. Interestingly, both qRT-PCR and sequence analysis in CDS region demonstrated that the candidate genes in present study might play important roles in rice Al tolerance.

**Conclusions:**

The present study provided important information for further using these elite genes existing in Ting’s core collection for improvement of rice Al tolerance.

## Background

Over 50% of the world’s arable land is acidic and about 13% of global rice were produced on acidic soils [1–3]. Rice (*Oryza sativa* L.) is one of the most important crops in the world, nearly half of the world’s population feed on rice. Aluminum (Al) which is the most abundant metal in the earth’s crust could be solubilized into trivalent Al (Al^3+^) in acidic soils when pH value is below 5.0. Root growth might be inhibited and rice yield could be reduced significantly when Al^3+^ is at high concentration [4, 5] while beneficial at low level [6]. Therefore, improving Al tolerance is significantly useful for rice production.

Uncovering genetic mechanism of Al tolerance in rice is the premise for Al tolerance improvement. Plant physiologists and breeders have been focusing on revealing genetic mechanism of Al tolerance in rice [7–17]. Two detoxification mechanisms under Al^3+^ toxicity were illuminated in above studies, i.e. called exclusion of Al^3+^ producing in root cells and excreting chelating chemicals [18] and accumulation of Al^3+^ called internal detoxification [10, 14, 19, 20].

Many quantitative trait loci (QTL) for rice Al tolerance had been reported in previous researches by using different mapping populations [9, 17, 21–25]. Furthermore, several genes related to Al tolerance in rice were cloned, i.e. *ART1* [14], *STAR1* [10], *STAR2* [10], *Nrat1* [13], *OsFRDL4* [18], *OsALS1* [26], *OsMGT1* [27], *ASR5* [20] and *ART2* [28]. We summarized above QTL/genes and found that these QTL/genes were not identical for the greater part due to different mapping population or Al toxicity concentration. Moreover, these also show that the genetic mechanism of Al tolerance in rice is great complicated and need more studies on it in the future.

There are several limitations of linkage mapping: 1. only two alleles at any given locus can be studied in bi-parental crosses; 2. high cost; 3. poor mapping resolution [29], whereas association mapping based on linkage disequilibrium (LD) could overcome these limitations [30] and enable researchers to use modern technologies to exploit natural variations [31]. A genome wide association study (GWAS) was reported in rice Al tolerance using about 36 thousand SNPs [9], and an association analysis was performed in rice Al tolerance in our previous study in which only 274 SSRs markers were used in association mapping [17]. However, no higher resolution GWAS on Al tolerance within natural population was performed in previous studies on Al tolerance in rice.

Ting’s rice collection which is one of the earliest rice collection in China consists of 150 varieties constructed from 2262 of 7128 original landraces [32]. Ting’s core collection had been used for association mapping on rice agronomic traits [33]. Moreover, Ting’s core collection had been reported that it possessed a wide-range of phenotypic variation for Al tolerance and was used to perform association mapping on Al tolerance [17]. Therefore, Ting’s core collection could be an appropriate population for GWAS on rice Al tolerance.

In the present study, a GWAS for rice Al tolerance based on relative root elongation (RRE) in seedling stage was carried out using Ting’s core collection with more than 3.8 million high quality SNPs. Candidate regions identified by GWAS were compared with regions identified as QTL in previous studies and with Al sensitive mutants and/or candidate genes. This study would provide important information of candidate genes for Al tolerance improvement in rice.

## Results

### Identification of phenotypic variations for Al tolerance

**Fig. 1:**
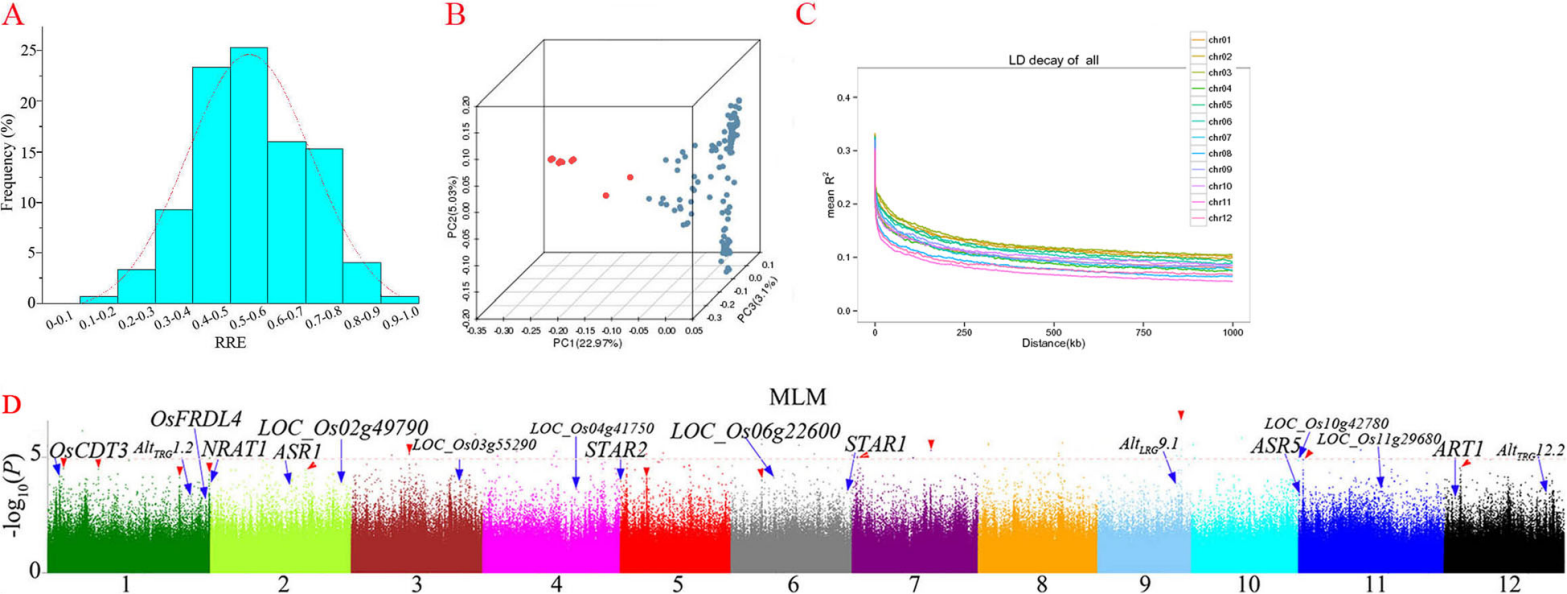
Phenotype, genetic dissection and GWAS for Al tolerance within Ting’s core collection. **a**. Frequency distribution of RRE in Ting’s core collection; **b**. Principal component analysis on 3.8 million SNPs of Ting’s core collection; **c**. Genome-wide average LD decay estimated in Ting’s core collection; **d**. Manhattan plots of MLM for RRE in GWAS. Negative log_10_(*P*) values from a genome-wide scan are plotted against position on each of 12 chromosomes. Blue and red arrow represent the loci close to previous genes as well as new loci, respectively. Red dash line represents significant threshold (*P* = 1.07 × 10^− 5^)

The rice landraces in Ting’s core collection revealed a wide range of phenotypic variation and indicated a normal distribution for Al tolerance which was identified by RRE (Fig. 1a, Additional file 5: Table S1) as well as showed strong Al tolerance with an average of RRE > 0.50 (Additional file 1: Figure S1).

### Genome re-sequencing and SNPs getting

Ting’s core collection was re-sequenced with whole genome re-sequencing method. High quality SNPs (Additional file 7: Table S3) distribution along position on each chromosome were shown in Additional file 2: Figure S2. 3,808,730 SNPs were generated totally, while there were 386,562 SNPs found in the CDS region (Additional file 7: Table S3).

### Population structure and LD estimation

PCA were performed with all SNPs for identifying the population structure of Ting’s core collection, and two subpopulations were observed (Fig. 1b). Moreover, we measured LD through *r*^*2*^ for Ting’s core collection using the SNP data. We found that LD dropped to half of its maximum value at 100~350 kb on 12 chromosomes (Fig. 1c).

### GWAS

A total of 3,808,730 SNPs were applied into GWAS for Al tolerance using GLM and MLM. We used *P* = 1.07 × 10^− 5^ and *P* = 2.63 × 10^− 7^ as the significant threshold for RRE in our study for MLM and GLM, respectively (Fig. 1d and Additional file 3: Figure S3). 5 and 38 significant loci distributing on 12 chromosomes were identified totally for Al tolerance in GLM and MLM, respectively (Table 1). The highest significant signal on each chromosome were shown in Table 1 in GLM and MLM. Our results indicated that the false positives were well controlled in this study (Additional file 4: Figure S4).

**Table 1:** Summary of association mapping results for relative root elongation using GLM and MLM

Chr.	Number of significant loci		SNP with the highest -log_10_*P* (IRGSP-1.0)	
	GLM (−log_10_*P* > 6.58)	MLM (−log_10_*P* > 4.97)	Position (GLM)	Position (MLM)
1	1	2	10,953,291 (−log_10_*P* = 6.7)	10,953,291 (−log_10_*P* = 5.6)
2	1	1	14,694,840 (−log_10_*P* = 7.2)	14,694,840 (−log_10_*P* = 5.0)
3	0	4	34,440,793 (−log_10_*P* = 6.5)	34,440,793 (−log_10_*P* = 5.5)
4	0	3	6,783,812 (−log_10_*P* = 5.9)	6,783,812 (−log_10_*P* = 5.1)
5	0	3	6,223,962 (−log_10_*P* = 6.1)	6,223,962 (−log_10_*P* = 5.1)
6	1	5	7,814,182 (−log_10_*P* = 7.2)	7,814,182 (−log_10_*P* = 5.3)
7	0	5	17,113,274 (−log_10_*P* = 6.1)	17,113,274 (−log_10_*P* = 5.1)
8	1	6	2,885,626 (−log_10_*P* = 6.8)	2,885,626 (−log_10_*P* = 5.1)
9	0	4	19,838,960 (−log_10_*P* = 6.5)	19,838,960 (−log_10_*P* = 6.0)
10	1	4	10,133,350 (−log_10_*P* = 6.8)	10,133,350 (−log_10_*P* = 5.5)
11	0	1	13,457,541 (−log_10_*P* = 6.1)	13,457,541 (−log_10_*P* = 5.1)
12	0	0	11,951,228 (−log_10_*P* = 6.0)	10,528,920 (−log_10_*P* = 4.5)
Total	5	38	–	–

In our study, we identified 17 genes relating to rice Al tolerance which were reported in previous studies (Fig. 1d) with a modest significant threshold (*P* < 0.001). Besides, 13 new candidate regions with high LD and 69 new candidate genes for Al tolerance were identified firstly using MLM in our study (Additional file 8: Table S4). New significant association regions with smaller *P* value and higher consecutive peak detected by MLM for Al tolerance were shown in Fig. 1d. And detailed distribution of these new gene-based association signals were included in Additional file 9: Table S5.

### Effects of allele variations

**Fig. 2:**
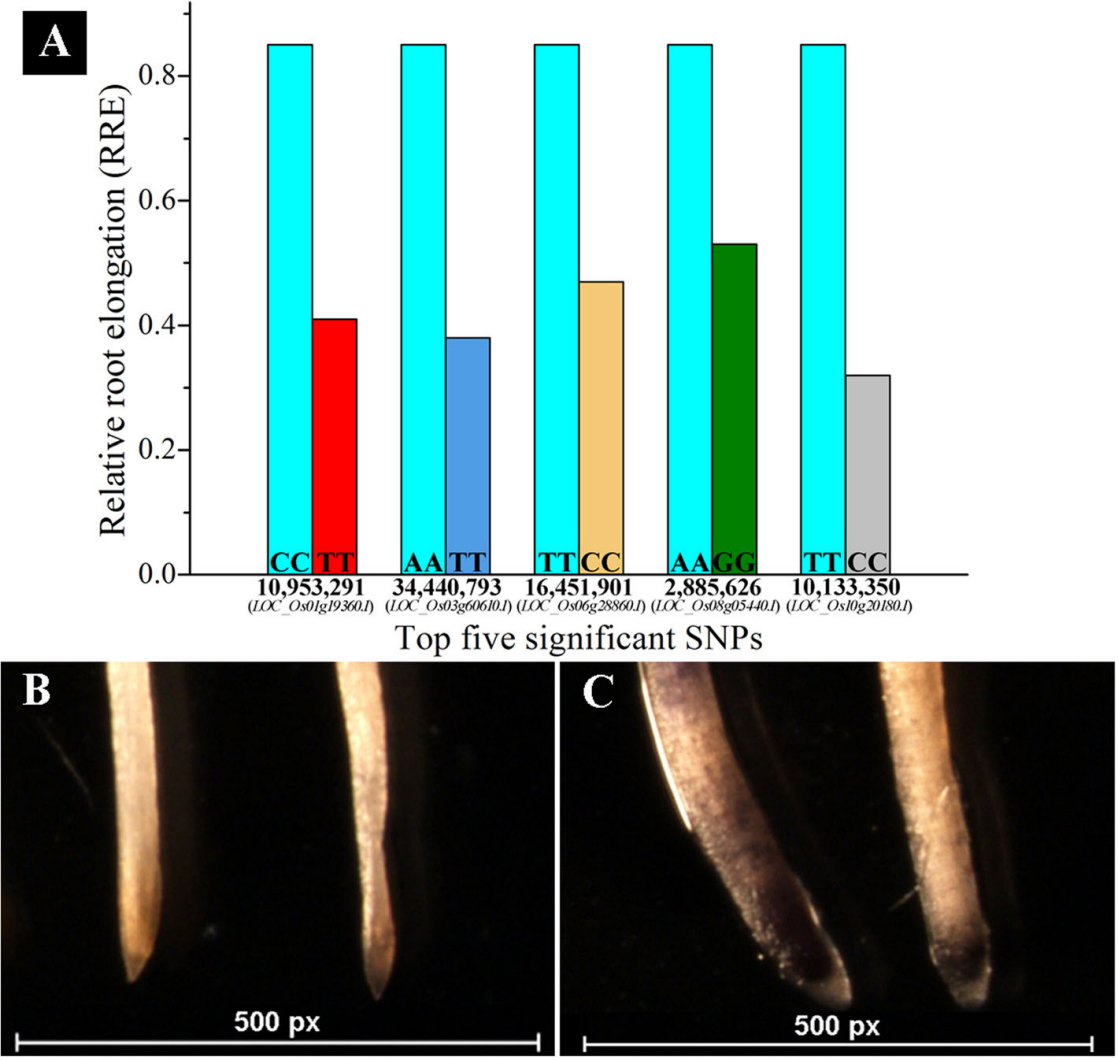
Effect analysis of allele variations on rice Al tolerance. **a**. RRE of varieties with reference and alternative allele at top 5 significant gene-based SNPs under Al toxic after 24 h. Sea blue bar represents RRE of reference allele (Nippobare), other five colors represent RRE of alternative alleles for top five significant SNPs; **b**. Root tips of variety with reference and alternative allele locating at 10,953,291 on chromosome 1 stained by hematoxylin without Al toxicity. Left root tip belongs to the variety (Youzhan) with alternative allele, while right belongs to reference allele (Nipponbare); **c**. Root tips of variety with reference and alternative allele locating at 10,953,291 on chromosome 1 stained by hematoxylin after 24 h under Al toxicity. Left root tip belongs to the variety (Youzhan) with alternative allele, while right belongs to reference allele (Nipponbare)

Allele analysis was performed for 13 high significant SNPs and frequency of alleles existing in Ting’s core collection were higher than those in reference (Table 2). Furthermore, top five significant SNPs at the gene-based location were selected for detecting the effects of allelic variations on Al tolerance (Table 2). RRE of varieties with alternative alleles for above five SNPs were not larger than those with reference (Nipponbare) alleles in Ting’s core collection (Fig. 2a). Moreover, staining by hematoxylin of Nipponbare (Al-tolerant) with allele C was lighter than Youzhan (an Al-sensitive variety) with allele T at 10,953,291 on chromosome 1 after Al toxicity treatment (Fig. 2b and c).

**Table 2:** Top highest genome-wide significant association signals of RRE using MLM

Trait	Chr.	Position (IRGSP-1.0)	Reference allele	Alternative allele	Alternative allele frequency	-log_10_(*P*)	Candidate/known gene^a^
RRE	1	10,953,291	C	T	0.87	5.58	*LOC_Os01g19360.1*
	2	14,694,840	A	G	0.85	5.04	
	3	34,440,793	A	T	0.88	5.45	*LOC_Os03g60610.1*
	6	7,814,182	A	G	0.73	5.24	
	6	7,814,184	C	T	0.74	5.24	
	6	7,814,207	C	T	0.75	5.06	
	6	16,451,901	T	C	0.86	5.02	*LOC_Os06g28860.1*
	8	18,696,483	T	C	0.67	5.11	
	8	2,885,626	A	G	0.63	5.10	*LOC_Os08g05440.1*
	8	2,885,642	A	T	0.61	5.04	
	9	19,838,960	G	A	0.88	5.99	
	10	10,133,350	T	C	0.58	5.30	*LOC_Os10g20180.1*
	10	8,299,577	T	C	0.71	5.03	

Note: ^a^Gene ID of MSU rice genome annotation project (http://rice.plantbiology.msu.edu/)

### Transcriptomic analysis

**Fig. 3:**
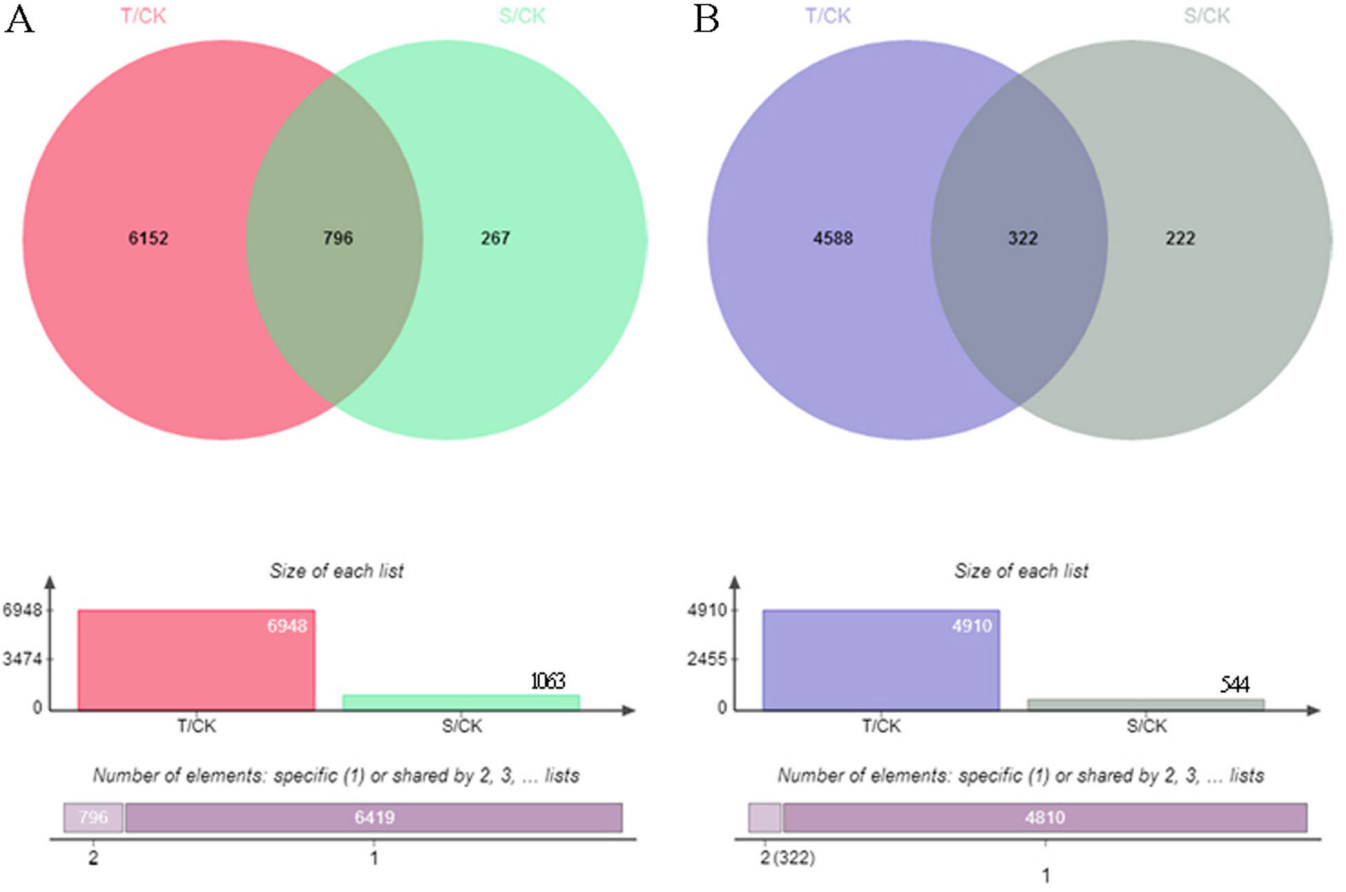
Venn diagram representing the number of differentially expressed genes between Al tolerant and sensitive variety. **a**. Up-regulated genes; **b**. Down-regulated genes. T, S and CK represent Al tolerant variety, Al sensitive variety and without Al toxic, respectively

6948 genes were up-regulated for Al tolerant variety between treatment under (T) and without (CK) Al toxicity, while 1063 genes were up-regulated for Al sensitive variety and totally 796 genes were up-regulated both in Al tolerant and sensitive varieties (Fig. 3a). There were 4910, 544 and 322 genes which were down-regulated for Al tolerant, sensitive and both, respectively (Fig. 3b). Among 69 candidate genes identified through GWAS, 20 genes were also detected with significant difference between treatment under and without Al toxicity by transcriptome sequencing (Additional file 10: Table S6).

### qRT-PCR and sequence analysis in CDS region

**Fig. 4:**
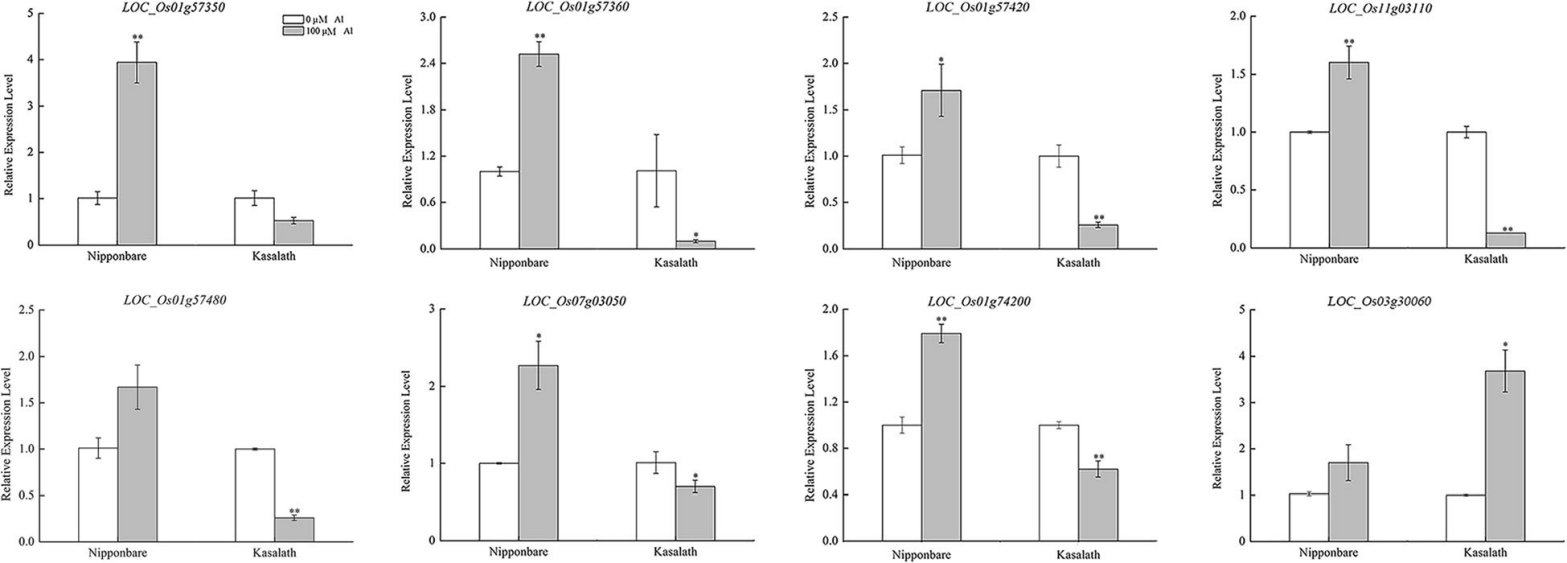
Expression of 8 candidate genes in rice root tip. * and ** represent significant difference in *p* < 0.05 and *p* < 0.01 level (T-test), respectively

The expression of eight in above candidate genes detected by transcriptome sequencing were significant different in root tip between Nipponbare (Al tolerant) and Kasalath (Al sensitive) (Fig. 4). At the meanwhile, 20 candidate genes were chosen for sequencing analysis in CDS region and sequence variations including substitutions, deletions and insertions could be found in seven genes CDS region between Al-tolerant and Al-sensitive varieties, which lead to amino acid variations (Table 3).

**Table 3:** Detection of frame shift mutation in varieties with different Al tolerance

Candidate gene	Varieties with different Al tolerance	Position from the initial codon in CDS region	Codon	Amino acid
*LOC_Os01g57350.1*	Nipponbare	182nd	CAG	Glutamine
	Kasalath		GAG	Glutamic acid
	Nipponbare	261st	TAC	Tyrosine
	Kasalath		CAC	Histidine
*LOC_Os03g30060.1*	Nipponbare	20th	AAG	Lysine
	Kasalath		CAG	Glutamine
	Nipponbare	66th	GCC	Alanine
	Kasalath		ACC	Threonine
	Nipponbare	88th	GTG	Valine
	Kasalath		ATG	Methionine
	Nipponbare	125th	GCA	Alanine
	Kasalath		GGA	Glycine
	Nipponbare	126th	GCG	Alanine
	Kasalath		TCG	Serine
	Nipponbare	155th	AAC	Asparagine
	Kasalath		AGC	Serine
	Nipponbare	184th	GCG	Alanine
	Kasalath		ACG	Threonine
	Nipponbare	220th	CAG	Glutamine
	Kasalath		GAG	Glutamic acid
	Nipponbare	246th	GCC	Alanine
	Kasalath		Deletion	Deletion
	Nipponbare	260th	ATG	Methionine
	Kasalath		GTG	Valine
	Nipponbare	276th	ATC	Isoleucine
	Kasalath		GTC	Valine
	Nipponbare	277th	GCG	Alanine
	Kasalath		ACG	Threonine
*LOC_Os07g03050.1*	Nipponbare	7th	TAT	Tyrosine
	Kasalath		TCT	Serine
	Nipponbare	344th	TGT	Cysteine
	Kasalath		AGT	Serine
*LOC_Os01g57360.1*	Nipponbare	32nd	AAG	Lysine
	IR64		AAT	Asparagine
	Nipponbare	348th	GGC	Glycine
	IR64		TGC	Cysteine
*LOC_Os11g03110.1*	Nipponbare	261st~269th	Deletion	Deletion
	Kasalath		Insertion	Insertion
	Nipponbare	608th	GTG	Valine
	Kasalath		GCG	Alanine
*LOC_Os01g74200.1*	Nipponbare	12th	TCG	Cysteine
	IR64		TCA	Serine
*LOC_Os09g33550.1*	Nipponbare	50th	CAC	Histidine
	IR64		CCC	Proline
	Nipponbare	58th	GCG	Alanine
	IR64		ACG	Threonine
	Nipponbare	65th	GCG	Alanine
	IR64		GTG	Valine

## Discussion

Al toxicity is one of the most limits for rice production. QTL mapping based on segregating population is the main method for most of previous studies for Al tolerance. Linkage mapping which were constructed using typical Al tolerant and sensitive varieties. However, only two alleles at any given locus can be studied in linkage mapping. In this study, Ting’s core collection consisted of rice landraces which represents an intermediate stage in domestication between wild and elite cultivars [34] was used for association mapping for Al tolerance. A wide-range of phenotypic variations and a normal distribution for Al tolerance were indicated in Ting’s core collection. Therefore, Ting’s core collection could be used as for GWAS on Al tolerance.

There were two researches on rice Al tolerance using low resolution GWAS [9, 17]. More than 3.8 million SNPs were applied into GWAS for Al tolerance in the present study and much higher in resolution than above two studies. Moreover, LD dropped to half of its maximum value at 100~350 kb (Fig. 1c) which is agreement with the previous measurements [35–37].

The research of Famoso et al. (2011) is the first GWAS on Al tolerance in rice [9]. In this study, 48 regions associated with Al tolerance were identified by GWAS. In total, three regions/loci were detected in both study of Famoso et al. (2011) and present. Ting’s core collection was used to perform GWAS in our previous study using 274 SSR markers [17], and 23 significant trait-marker associations were discovered. However, no identical region/locus was identified in both our previous and present study. There might be three reasons for explaining this: 1. Different markers with distinct resolutions used in two researches; 2. No identical significant threshold used in two researches. In our previous study, *P* < 0.05 was applied to judge a significant region/locus. The significant trait-marker associations might not be detected in present study; 3. RRE of 18 varieties were missing in present study while there were only three missing in our previous studies.

In the present study, we found that there was no signals with a strong significant threshold in previously reported genes or regions. It is probably because: 1. Different materials were used in previous studies; 2. No same statistical model was used in previous studies; 3. Most of QTL were identified with mutations in previous studies; 4. Al tolerance were affected by numerous alleles with minor effects, so previous QTL/genes cannot be detected in the present study. Thus, a modest significant threshold (*P* < 0.001) was used in the present study which is same to the study of Huang et al. (2015) for grain number [38]. We identified 17 genes (*P* < 0.001) relating to rice Al tolerance which were reported in previous studies (Fig. 1d). Significant association signals close to *OsCDT3* on chromosome 1 [39], *Alt*_*TRG*_*1.2* on chromosome 1 [9], *OsFRDL4* on chromosome 1 [15], *NRAT1* on chromosome 2 [13], *ASR1* on chromosome 2 [7], *LOC_Os02g49790* on chromosome 2 (Rice Genome Annotation Project, RGAP), *LOC_Os03g55290* on chromosome 3 (RGAP), *LOC_Os04g41750* on chromosome 4 (RGAP), *STAR2* on chromosome 5 [10], *LOC_Os06g22600* on chromosome 6 (RGAP), *STAR1* on chromosome 6 [10], *Alt*_*LRG*_*9.1* on chromosome 9 [9], *LOC_Os10g42780* on chromosome 10 (RGAP), *ASR5* on chromosome 11 [20], *LOC_Os11g29680* on chromosome 11 (RGAP), *ART1* on chromosome 12 [14] and *Alt*_*TRG*_*12.2* on chromosome 12 [9] were detected in our study. It is necessary to notice that –log_10_(*P*) value of these significant signals both in our study and the first as well as the only one GWAS research [9] for rice Al tolerance are not large as those in GWAS researches of other rice traits. In our opinion, this might be due to high complexity of rice Al tolerance mechanism.

In our opinion, those peaks with consecutive loci which are below the significant threshold (−log10(*P*) = 4.97) are also valuable to be analyzed deeply because the reported genes for Al tolerance are below 4.97, hence, we set -log10(*P*) = 3.0 as the threshold for above peaks. 13 new candidate regions with high LD and 69 new candidate genes (Additional file 8: Table S4) for Al tolerance were identified firstly using MLM in our study. Among 69 candidate genes for Al tolerance, 48 genes could be not predicted their performance on Al detoxification through annotation, while the remaining 21 genes could be speculated their detoxifying Al mechanism with annotation information and previous studies (Additional file 8: Table S4). For instance, *LOC_Os01g09370.1* encodes an ankyrin repeat domain-containing protein 28, and ankyrin repeat domain-containing protein was reported that it is probably involved in the regulation of antioxidation metabolism shared by stress responses [40]. Therefore, *LOC_Os01g09370.1* might be one gene which is related to Al stress. *LOC_Os01g57350.1*, *LOC_Os01g57360.1*, *LOC_Os01g57420.1* and *LOC_Os07g29220.1* encode a diacylglycerol kinase, an acyltransferase, a diacylglycerol kinase as well as a cyclopropane-fatty-acyl-phospholipid synthase respectively which are necessary to lipid metabolism in plant [41]. Moreover, lipids were reported that they might participate in rice Al detoxification [42, 43]. Interestingly, few scientists paid attention to study on lipid in rice Al tolerance. Pentatricopeptide repeat domain containing protein encoded by *LOC_Os01g57410.1* was identified that this protein was a regulator for abiotic stress [44]. *LOC_Os01g25090.1*, *LOC_Os01g74160.1* and *LOC_Os03g30080.1* encode a transposon protein, putative, CACTA, En/Spm sub-class [45], a carboxyl-terminal peptidase [46] as well as a transposon protein, putative, CACTA, En/Spm sub-class [44] which were associated with metal accumulation. *LOC_Os09g33559.1* encodes a proline-rich family protein, while proline was reported it relating to Al metabolism [47].

To further test positive capability of candidate genes identified in our study, we chose two varieties (Al tolerant and sensitive) to perform transcriptome sequencing. Among 69 candidate genes identified through GWAS, 20 genes were also detected with significant differences between treatments under and without Al toxicity by transcriptome sequencing (Additional file 10: Table S6). There were three candidate genes (i.e. *LOC_Os01g57350.1*, *LOC_Os01g57420.1* and *LOC_Os07g29220.1*) which were up regulated (T versus CK) obviously in Al tolerant variety while no significant difference in Al sensitive variety among candidate genes in group lipid metabolism, and another candidate gene (i.e. *LOC_Os01g57360.1*) was up regulated in Al sensitive variety. Both *LOC_Os01g57450.1* and *LOC_Os01g57480.1* which are in group abiotic stress response were up regulated in Al tolerant variety while no significant difference in Al sensitive variety. In group membrane protein, both *LOC_Os01g74180.1* and *LOC_Os06g14030.1* were up regulated in Al tolerant variety while no significant difference in Al sensitive variety. Expression of candidate gene *LOC_Os09g33559.1* was down regulated in Al tolerant variety while no significant difference in Al sensitive variety. Moreover, remaining 11 candidate genes in group unknown were up or down regulated for two varieties. Furthermore, qRT-PCR and sequence analysis in CDS region also gave more supports to our candidate genes.

In addition, after GWAS experiment, we agree that it is a better way to carry out further studies by using varieties with extreme values in Ting’s core collection. In present study, after GWAS transcriptome analysis, histological observation, sequence comparisons of CDS and qRT-PCR were used to support the results of GWAS. Histological observation was performed to identify the effects of allele variations of significant SNPs from GWAS. Transcriptome analysis was to try to find some identical genes to GWAS. Both sequence comparisons of CDS and qRT-PCR were designed to try to support the candidate genes identified by GWAS and transcriptome. We chose different materials for further study because the candidate genes in present study were supposed to be supported by different materials. And we tried to prove the candidate genes from GWAS which are natural variations exiting in Ting’s core collection would have effects to rice Al tolerance in other materials.

## Conclusions

In this study, 69 new candidate genes for Al tolerance were identified using GWAS and some among 69 candidate genes were also detected through transcriptome sequencing, qRT-PCR and checking of sequence variation of CDS. It is worth to perform deeper research on them.

## Methods

### Plant material

Ting’s core collection with 150 rice landraces [48] was used in present study. The information of 150 landraces are shown in Additional file 5: Table S1.

### Phenotyping for Al tolerance

Seeds of Ting’s core collection were harvested from the farm of China National Rice Research Institute, Hangzhou (30°3 N, 120°2E), during the late season (May–October) in 2016. Relative root elongation (RRE) was used to evaluate Al tolerance of all varieties, please see more details in our previous study [17]. RRE of 10 seedlings of each variety growing in parallel were measured with a ruler before and after the treatments (24 h) in one replicate, and six replications were performed. Fu et al. (2010) referenced that RRE ≥ 0.5 should be used as a criterion to find out Al tolerant varieties [49].

The root tips (1~2 cm) of rice seedlings cultivated for 24 h in the presence or absence of Al were gently shaken in a 0.5 mmol l^− 1^ CaCl_2_ (pH = 4.0) solution for 10 min. The root tips were submerged into hematoxylin solution (0.2% hematoxylin and 0.02% potassium iodide). After 45 min, the root tips were soaked into deionized water till no clear color on root tips. Then the root tips were photographed using stereoscopic and light microscopes. We chose Youzhan as one of the varieties which had different alleles (alternative allele) to Nipponbare (reference allele) for some SNPs to be stained by hematoxylin solution.

### Genome re-sequencing and SNP calling

Genomic DNA from a single plant was used for sequencing. Re-sequencing was performed by Illumina HiSeq™ 4000 with 6~7 folds of genome coverage. We performed the mapping to the rice reference genome (IRGSP-1.0) for the 150-bp reads by using bwamem with the –M option of BWA software [50], and the mapped reads were realigned by using RealignerTargetCreator in GATK [51]. SNPs were labeled with the −glm BOTH option of UnifiedGenotyper in GATK. After filtering the SNPs with low minor-allele frequency (5%), a total of 3,808,730 SNPs was remained for GWAS in our study. Please read more details in our previous study [52].

### Genetic analyses

Genetic analyses were performed following our previous study [52]. Principal component analysis (PCA), and LD decay analysis among Ting’s core collection were performed based on all SNPs. SPSS 17.0 was used for PCA analysis. The LD was evaluated using squared Pearson’s correlation coefficient (*r*^*2*^) calculated with the −*r*^*2*^ command of the software PLINK [53]. The Q-matrix was obtained with the membership probability of each variety using ADMIXTURE Version 1.22. The Loiselle algorithm was chosen for calculating kinship matrix by software SPAGeDi [54].

### GWAS

A total of 3,808,730 SNPs were used for GWAS. General linear model (GLM, without Q-matrix) and mixed linear model (MLM, K + Q) were performed using TASSEL software (www.maizegenetics.net/tassel). *P* ≤ 2.63 × 10^− 7^ (*P* = 1/n; n = total markers, which is a rough Bonferroni correction corresponding to -log_10_(*P*) = 6.58) should be defined as the suggestive threshold which is same to that in our previous study [52] in present study, but significant locus can not be identified according to this threshold. Thus, another significance threshold was calculated, i.e., a minimum Bayes factor (mBF). The mBF was calculated using the following formula: mBF  =   − e_*_P_*_ln(*P*) [55], thus, the significance threshold in present study was -log10(*P*) = 4.97.

### Transcriptomic analysis

Bashizi, one Al-tolerant variety (RRE = 0.65) and Heikedanuo, one Al-sensitive (RRE = 0.34) from Ting’s core collection (Additional file 5: Table S1) were chosen for transcriptome analysis. Total RNA of root tip after one day under 100 μmol l^− 1^ AlCl_3_ was extracted with the miRNeasy Kit (QIAGEN, USA). The RNA samples were evaluated on agarose gels, quantified in a spectrophotometer and stored at − 80 °C. RNA sequencing was performed by Illumina NextSeq 500 for 2 × 125-bp paired-end sequencing. Samples under Al toxic treatment and without Al toxic in three times as well as one time respectively were collected for RNA sequencing [56].

### Real-time qRT-PCR

Nipponbare (RRE = 0.85), one well known Al-tolerant variety and Kasalath (RRE = 0.30), one well known Al-sensitive were chosen for real-time qRT-PCR analysis. Total RNA of root tip after one day without and under 100 μmol l^− 1^ AlCl_3_ was extracted with the miRNeasy Kit (QIAGEN, USA). RNA was converted to cDNA using the protocol supplied by the manufacturer of ReverTra Ace qPCR RT Master Mix with gDNA remover (TOYOBO). The expression was determined with THUNDERBIRD™ SYBR® qPCR Mix without ROX (TOYOBO) by Roche Light Cycler® 480II. The primer sequences for qRT-PCR were listed in Additional file 6: Table S2. Ubiquitin was used as an internal control. Relative expression levels were calculated by 2^-ΔΔCt^ method. Three independent biological replicates were made for each treatment. The volume of the qRT-PCR reaction system was 10 μl: SYBR Premix Ex Taq II, 2x, 5 μl; PCR Primer (Forward+Reverse, 10uM), 2 μl; cDNA, 2 μl; ddH_2_O, 1 μl. The profile of PCR program is: 95 °C for 3 min; 45 cycles of 95 °C for 10 s, 58 °C for 15 s, 72 °C for 25 s; 95 °C 10 s; 65 °C 1 min; 40 °C 1 min.

### CDS sequence variation analysis of candidate genes

Nipponbare, one well known Al-tolerant variety. Kasalath and IR64 (RRE = 0.29), two well known Al-sensitive varieties were chosen for sequence variation analysis. Genomic DNA was extracted using modified SDS method. Several primers were designed for CDS sequencing of each gene. Then, CDS were amplified by PCR, the volume of the PCR reaction system was 10 μl. The profile of PCR program is: 94 °C for 5mins; 31 cycles of 94 °C for 1 min, 52–58 °C for 1 min, 72 °C for 1–2 min; and a 5mins final extension at 72 °C. Amplified products were size separated by 1% agrose gel electrophoresis and purified using QIAquick Gel Extraction Kit (QIAGEN, Germantown, MD, USA). After sequencing of PCR products, sequences of same CDS were assembled and CDS sequences of Nipponbare, Kasalath and IR64 were aligned using software BioEdit.

## Supplementary information

**Additional file 1: Figure S1.** Box chart of Al tolerance (RRE) in Ting’s core collection.

**Additional file 2: Figure S2.** SNP and Indel distribution along position in each chromosome. Longitudinal axis represent the number of SNP and Indel.

**Additional file 3: Figure S3.** Manhattan plots of GLM for RRE in GWAS. Negative log_10_(*P*) values from a genome-wide scan are plotted against position on each of 12 chromosomes. Red dash line represents significant threshold (*P* = 1 × 10^− 5^).

**Additional file 4: Figure S4.** Plots of observed versus expected *P*-values using MLM and GLM for RRE. A. MLM; B. GLM. Red symbol represents expected *P*-values, and Blue symbol represents observed *P*-values.

**Additional file 5: Table S1.** Accessions, variety names, origin and RRE of 150 rice varieties in Ting’s core collection.

**Additional file 6: Table S2.** Primers used in qRT-PCR in this study.

**Additional file 7: Table S3.** Summary of categorized SNPs and Indels.

**Additional file 8: Table S4.** List of new loci in association analysis of root relative elongation under Al toxic.

**Additional file 9: Table S5.** List of all *P* value ranked genes in the gene-based association analysis of root relative elongation using MLM.

**Additional file 10: Table S6.** List of new gene’s expression in association analysis of root relative elongation under Al toxic and without Al toxic.

## Data Availability

The datasets used during the current study are available from the corresponding author on reasonable request.

## Abbreviations

CDS: Coding sequence
DNA: Deoxyribonucleic acid
EMMAX: Efficient mixed model association eXpedited
IRGSP: International Rice Genome Sequencing Project
qRT-PCR: Quantitative reverse transcription-PCR
QTL: Quantitative trait locus
SDS: Sodium dodecyl sulfate
SNPs: Single Nucleotide Polymorphisms
SSR: Simple Sequence Repeats
