# Supplementary figures and images for: Mining candidate gene for rice aluminum tolerance through genome wide association study and transcriptomic analysis
Source: BMC Plant Biol. 2019 Nov 12;19:490. doi: 10.1186/s12870-019-2036-z (PMC6852983; doi:10.1186/s12870-019-2036-z)

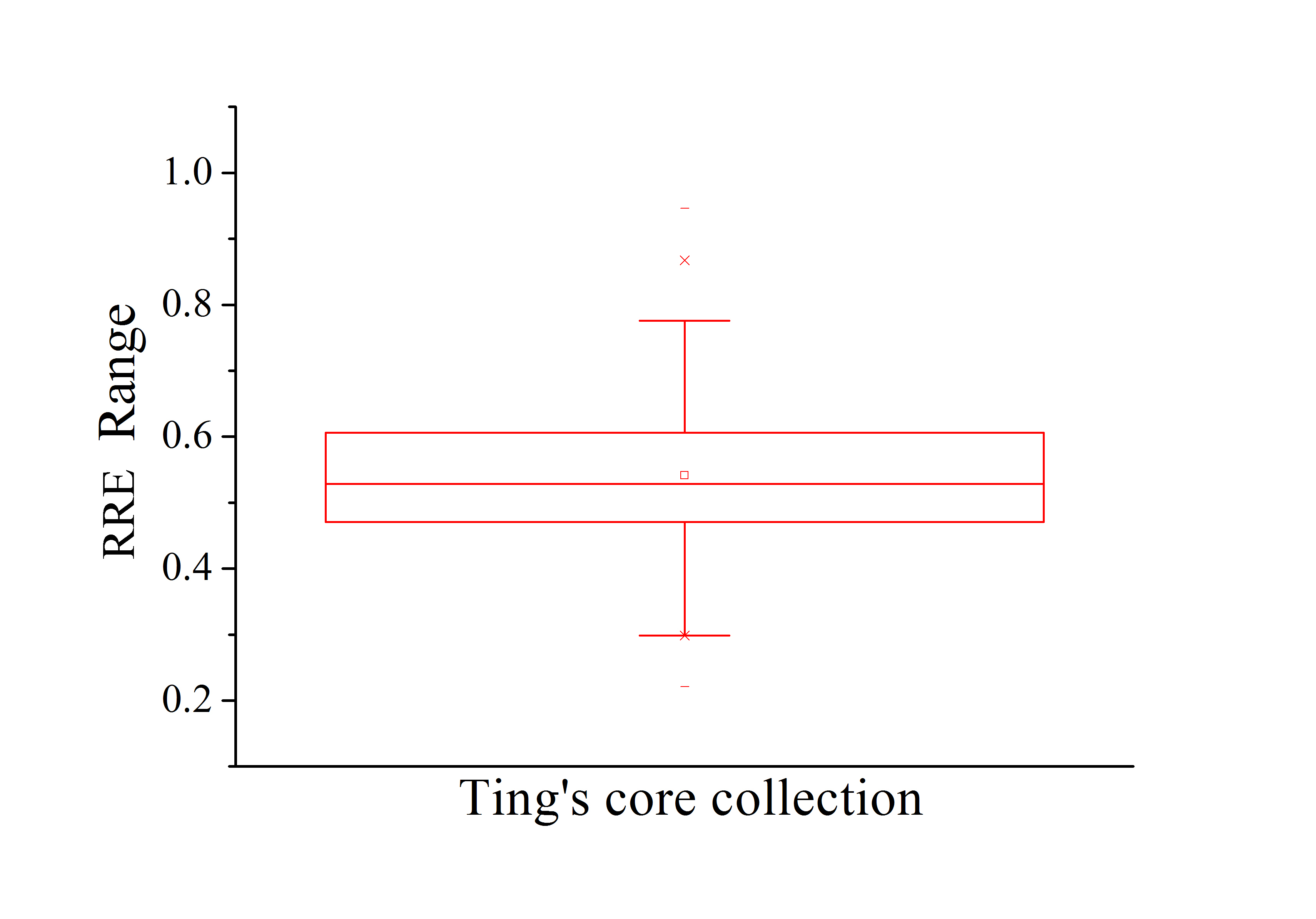

Supplement: Supplementary file 1 — Additional file 1: Figure S1. Box chart of Al tolerance (RRE) in Ting’s core collection. [file 12870_2019_2036_MOESM1_ESM.jpg]

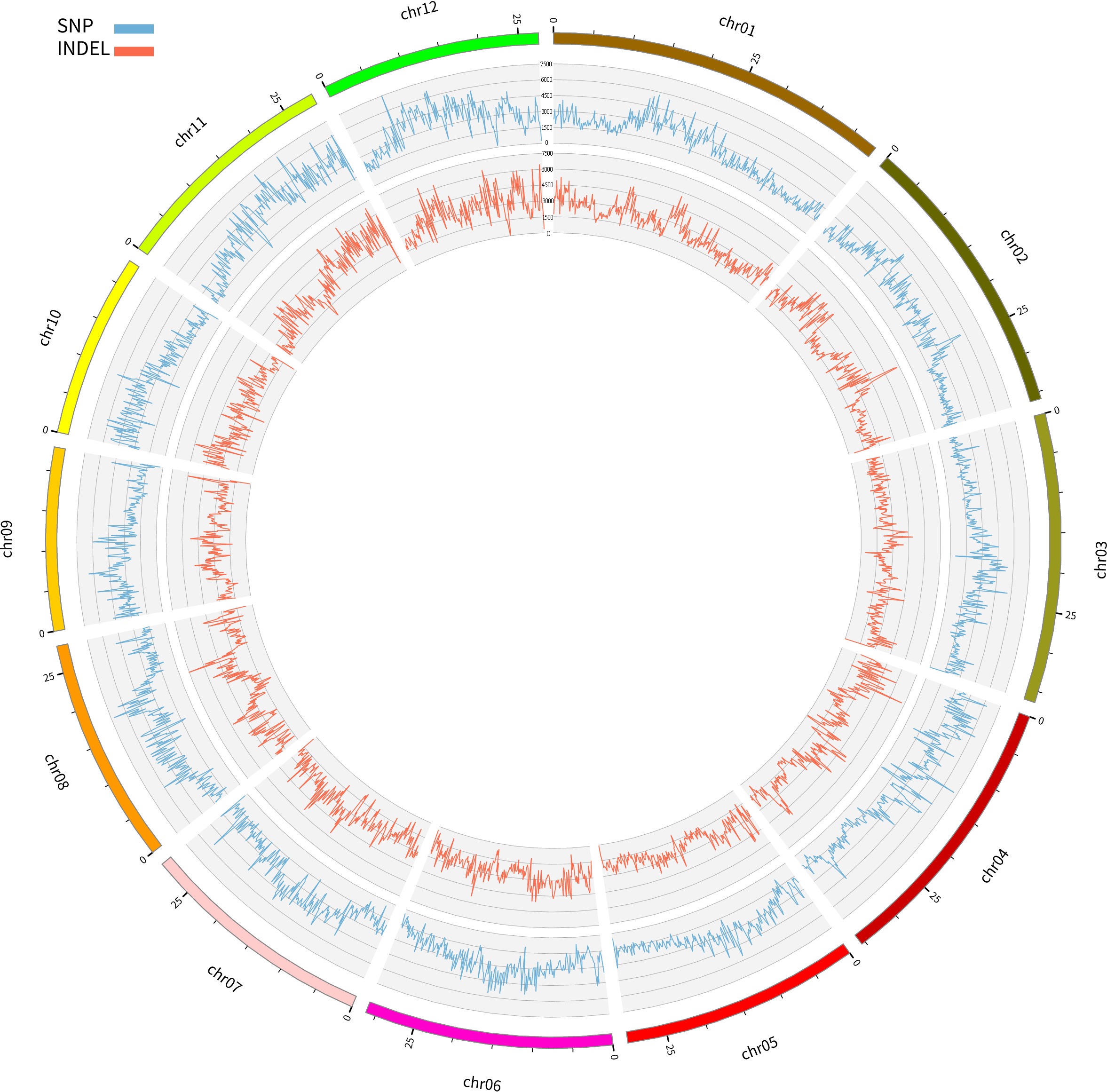

Supplement: Supplementary file 2 — Additional file 2: Figure S2. SNP and Indel distribution along position in each chromosome. Longitudinal axis represent the number of SNP and Indel. [file 12870_2019_2036_MOESM2_ESM.png]

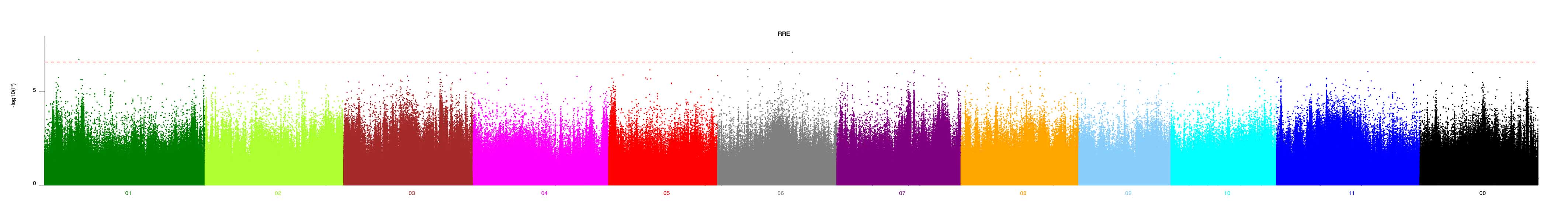

Supplement: Supplementary file 3 — Additional file 3: Figure S3. Manhattan plots of GLM for RRE in GWAS. Negative log10(P) values from a genome-wide scan are plotted against position on each of 12 chromosomes. Red dash line represents significant threshold (P = 1 × 10− 5). [file 12870_2019_2036_MOESM3_ESM.jpg]

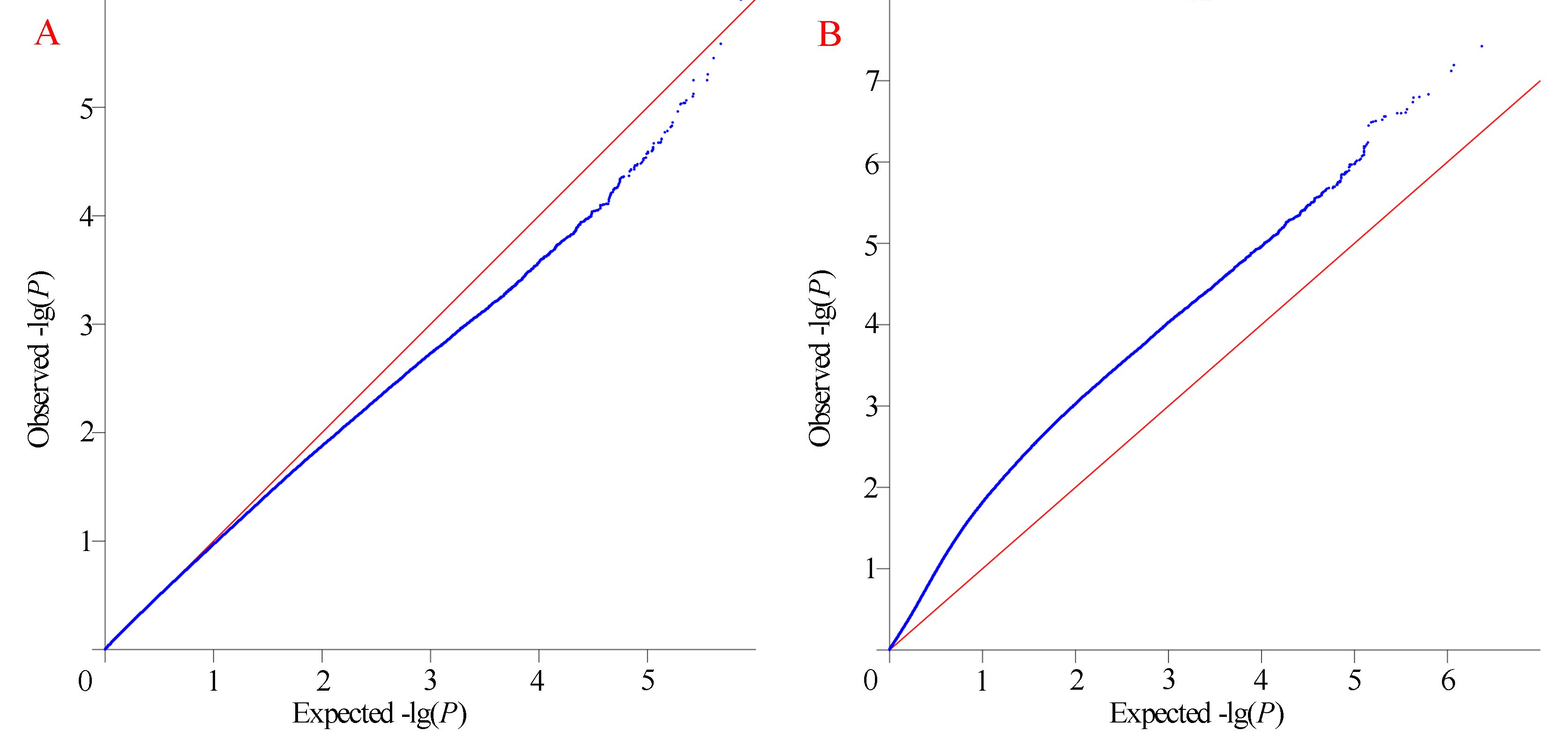

Supplement: Supplementary file 4 — Additional file 4: Figure S4. Plots of observed versus expected P-values using MLM and GLM for RRE. A. MLM; B. GLM. Red symbol represents expected P-values, and Blue symbol represents observed P-values. [file 12870_2019_2036_MOESM4_ESM.jpg]
