# Supplementary material for: Mining candidate gene for rice aluminum tolerance through genome wide association study and transcriptomic analysis
Source: BMC Plant Biol. 2019 Nov 12;19:490. doi: 10.1186/s12870-019-2036-z (PMC6852983; doi:10.1186/s12870-019-2036-z)
Supplement: Supplementary file 5 — Additional file 5: Table S1. Accessions, variety names, origin and RRE of 150 rice varieties in Ting’s core collection. [file 12870_2019_2036_MOESM5_ESM.docx]

**Additional file 5: Table S1** Accessions, variety names, origin and RRE of 150 rice varieties in Ting’s core collection

| Acc. | Variety name | Origin | Relative root elongation (RRE)±S.D. | Acc. | Variety name | Origin | Relative root elongation (RRE)±S.D. |
| --- | --- | --- | --- | --- | --- | --- | --- |
| CC1 | Yin guang | Japan | 0.74±0.07 | CC81 | Dong an hou zi pu xiao he | Central China | 0.37±0.02 |
| CC3 | Ai you | Japan | 0.95±0.11 | CC82 | Tie gu pao | Central China | 0.33±0.05 |
| CC6 | Ben dao | North China | 0.84±0.27 | CC83 | Chi mao zhan | South China | 0.55±0.12 |
| CC9 | Jiu yue han | Northeast China | 0.55±0.02 | CC84 | Hu bei zao | Central China | 0.61±0.07 |
| CC12 | Gui zao bai he | Yangtze River region | 0.44±0.14 | CC85 | Ya jing mi | South China | 0.53±0.05 |
| CC13 | Xiang dao | North China | 0.51±0.20 | CC86 | Ba shi zi | Central China | 0.60±0.15 |
| CC14 | Zi jin gu | Northeast China | 0.76±0.15 | CC88 | Early | Unknown | 0.50±0.05 |
| CC16 | Nagabo | Taiwan | 0.52±0.17 | CC89 | Nuo | South China | 0.68±0.10 |
| CC17 | Bai ke da nuo | South China | 0.49±0.14 | CC91 | Hei nuo | Unknown | 0.39±0.08 |
| CC18 | San pai zhong | South China | 0.50±0.11 | CC92 | Da yi mao | Central China | 0.48±0.05 |
| CC19 | Kai xuan | Japan | 0.38±0.11 | CC93 | Gai cao zhan | Central China | 0.54±0.09 |
| CC20 | Shi ban zhan | North China | 0.85±0.02 | CC94 | Gamal | Unknown | 0.54±0.04 |
| CC21 | Hei ke da nuo | South China | 0.34±0.05 | CC95 | Bu gou wei | South China | 0.61±0.05 |
| CC22 | Shen shui wan dao | Yangtze River region | 0.64±0.21 | CC96 | Bai ke xi nuo | South China | 0.76±0.19 |
| CC23 | Hong ben dao | Yangtze River region | 0.55±0.05 | CC97 | Ben dao | North China | 0.84±0.27 |
| CC24 | Duan mang zi jin gu | Northeast China | 0.56±0.12 | CC98 | Ba xian shu | Japan | 0.61±0.14 |
| CC25 | Bei jing jiang mi | North China | 0.58±0.11 | CC99 | Guang hong mi dao | Yangtze River region | 0.50±0.02 |
| CC26 | Daeri | Celebes | 0.56±0.07 | CC100 | Wu mang yan guo qing | North China | 0.31±0.05 |
| CC27 | Jian tou nuo | South China | 0.60±0.12 | CC101 | Chang xu nuo | South China | 0.54±0.01 |
| CC28 | Long you man dao | Yangtze River region | 0.34±0.07 | CC102 | Jiang wan 15 | Central China | 0.50±0.17 |
| CC29 | Kun shan zhu zhou dao | Yangtze River region | 0.79±0.07 | CC103 | Bai hua er | South China | 0.60±0.02 |
| CC30 | Huang ke zao 2 | Yangtze River region | 0.41±0.06 | CC104 | Liu chang xian | South China | 0.55±0.17 |
| CC31 | Sheng fang da bai gu | North China | 0.58±0.04 | CC105 | Bai yin 3 | South China | 0.60±0.05 |
| CC32 | Xiao dou | Japan | 0.65±0.08 | CC106 | Shui zao huang pi | South China | 0.59±0.18 |
| CC33 | Poetih | Celebes | 0.71±0.18 | CC107 | Yin 2 dong 7 | South China | 0.67±0.14 |
| CC34 | Tebaro | Sumbawa | 0.51±0.18 | CC108 | Hou ma | South China | 0.48±0.05 |
| CC35 | Ao hua da gui tou hong | Yangtze River region | 0.54±0.04 | CC109 | Dong zhu 2 | South China | 0.53±0.18 |
| CC36 | Hui bei zi | Yunnan-Kweichow Plateau | 0.47±0.14 | CC110 | Hong gen da mi | South China | 0.38±0.07 |
| CC37 | Ba shi zi | Yangtze River region | 0.65±0.07 | CC111 | Ben cheng guan yin zhan | Central China | 0.56±0.11 |
| CC38 | Zao sheng da ye | Japan | 0.48±0.09 | CC112 | Xi miao gu | South China | 0.54±0.07 |
| CC39 | Bnlastog | Low latitude region | 0.72±0.21 | CC113 | 186-zao guan yin zhan | Central China | 0.64±0.08 |
| CC40 | Nuo mi | North China | 0.45±0.11 | CC114 | Chang mang hei ma zao | Yunnan | 0.52±0.07 |
| CC41 | Xi chuan huang liu | South China | 0.54±0.15 | CC115 | Shui tian zhan gu nuo | South China | 0.53±0.05 |
| CC42 | Hei ju dao | Yangtze River region | 0.40±0.11 | CC116 | Chang han da hua ke | South China | 0.49±0.02 |
| CC43 | Guang fuⅠ | Taiwan | 0.47±0.07 | CC117 | Da he | South China | 0.59±0.14 |
| CC44 | Zhong qi jia qing | Yangtze River region | 0.47±0.11 | CC118 | Zeng cheng hei nuo | South China | 0.72±0.11 |
| CC45 | Ⅲ-49-4xi chuan huang | Taiwan | 0.70±0.17 | CC119 | Mao he | South China | 0.44±0.11 |
| CC47 | Da liu tiao dao | Yangtze River region | 0.60±0.01 | CC120 | Bai gu zhan | Central China | 0.42±0.12 |
| CC49 | Chuan chi 1 | Central China | 0.34±0.05 | CC121 | Die zhi | South China | 0.52±0.04 |
| CC50 | Tai nong 46 | Taiwan | 0.49±0.04 | CC122 | You zhan | South China | 0.41±0.08 |
| CC51 | Ba chong sui | Japan | 0.36±0.11 | CC123 | Chang sha wu qu wan dao | Central China | 0.57±0.01 |
| CC52 | Yun nan bai | Central China | 0.62±0.05 | CC124 | Tong ling hu nan xian | Central China | 0.48±0.18 |
| CC53 | Liao yang ben di 4 | Northeast China | 0.55±0.25 | CC125 | Xiao mao dao | Central China | 0.55±0.08 |
| CC54 | You zhan hong | South China | 0.54±0.14 | CC126 | Jing xian si qu er gan | Central China | 0.40±0.17 |
| CC55 | Hei nuo | South China | 0.37±0.07 | CC127 | Zi xing er qu si dou xu | Central China | 0.54±0.13 |
| CC56 | Xian zi zhan | Central China | 0.40±0.11 | CC128 | Chen hui fu dao | Central China | 0.30±0.04 |
| CC58 | Chi bai gan zhan | Central China | 0.30±0.12 | CC129 | Bai gan zi | Central China | 0.38±0.15 |
| CC59 | Zeng cheng xiang shan zhan | South China | 0.52±0.11 | CC130 | Han lu wei zhan | Central China | 0.53±0.11 |
| CC60 | Cang wu shan he zhan | South China | 0.55±0.15 | CC131 | Xin hua san qu tang mao zhan | Central China | 0.47±0.02 |
| CC61 | Da gu zao | South China | 0.59±0.07 | CC132 | Ta gu zhan | Central China | 0.52±0.03 |
| CC62 | Jie yang dong liao zhong | South China | 0.70±0.25 | CC133 | Mian tiao zhan | Central China | 0.41±0.05 |
| CC63 | Nan xiong ku gua zao | South China | 0.54±0.05 | CC134 | Hu guang zhan | Central China | 0.47±0.08 |
| CC64 | Chang mang | South China | 0.64±0.15 | CC135 | Jiang an da ye zao | Central China | 0.41±0.02 |
| CC65 | Mandi | Celebes | 0.48±0.08 | CC137 | Da gu zao | Central China | 0.47±0.02 |
| CC67 | Hong zao gu | Yunnan-Kweichow Plateau | 0.56±0.08 | CC138 | Ding nan dong zhan | Central China | 0.45±0.07 |
| CC68 | Cang wu shan he zhan | South China | 0.55±0.15 | CC139 | Lao wu gu | Central China | 0.62±0.01 |
| CC69 | Zao die zhan gu | Central China | 0.52±0.11 | CC140 | Xin ban chang ke zi | Central China | 0.59±0.11 |
| CC70 | Su zhou zhan | Central China | 0.62±0.14 | CC141 | Bai zhan gu | Central China | 0.46±0.12 |
| CC71 | Yang zhan 3 | South China | 0.55±0.04 | CC142 | Si chuan zhan | Central China | 0.58±0.07 |
| CC72 | Luo ding zhan 1 | South China | 0.62±0.18 | CC143 | Gao jiao gui hua | Central China | 0.59±0.03 |
| CC73 | Gen yin 29 | South China | 0.77±0.19 | CC144 | Chang shu wu xi dao | Central China | 0.51±0.04 |
| CC75 | Hua bai ke | South China | 0.49±0.01 | CC145 | Da nuo bai dong | Central China | 0.59±0.12 |
| CC76 | Guang ye hong mi | South China | 0.52±0.05 | CC146 | Lin chuan da ye zao | South China | 0.69±0.03 |
| CC77 | Da nuo | South China | 0.64±0.07 | CC147 | Da bai cao | North China | 0.46±0.11 |
| CC78 | Bai xu | South China | 0.44±0.01 | CC148 | Chang ning wu qu nan tou zhan | Central China | 0.27±0.04 |
| CC79 | Mao he | South China | 0.44±0.10 | CC149 | Pi xian da ye zi | Central China | 0.45±0.11 |
| CC80 | Xu zai | South China | 0.36±0.07 |  |  |  |  |
