# Supplementary material for: Mining candidate gene for rice aluminum tolerance through genome wide association study and transcriptomic analysis
Source: BMC Plant Biol. 2019 Nov 12;19:490. doi: 10.1186/s12870-019-2036-z (PMC6852983; doi:10.1186/s12870-019-2036-z)
Supplement: Supplementary file 6 — Additional file 6: Table S2. Primers used in qRT-PCR in this study. [file 12870_2019_2036_MOESM6_ESM.docx]

**Additional file 6: Table S2** Primers used in qRT-PCR in this study

| **Gene** | **Forward sequence (5' -> 3')** | **Reverse sequence (5' -> 3')** |
| --- | --- | --- |
| Ubiquitin | TGGTCAGTAATCAGCCAGTTTGG | GCACCACAAATACTTGACGAACAG |
| *LOC_Os01g57350* | GTACAGGAAATGGCATGGCT | CTTCCTTCCCTGCACAATGG |
| *LOC_Os01g57360* | GGCTAGTCAGCGTGCATTAC | CCAGTCAACCTCAGTCCTGT |
| *LOC_Os01g57420* | GCAACCGACCATGCTTACAT | GCCTCATGGATGCCTTTAGC |
| *LOC_Os01g57480* | TTGGATGGCGTGTGTGATTC | CCTGCTTCTTCACTCTCCCA |
| *LOC_Os01g74200* | TGGCCGAAGTGAACTACCTT | CCGAGGCATGAACTCGTAGA |
| *LOC_Os03g30060* | GATTTACGGGTGGCTGGTTG | CGAGGCTTCTACCGTGTACT |
| *LOC_Os07g03050* | TGGAGATGGCGCAAATCAAG | GCTGTTCCACCTCCTCTTCT |
| *LOC_Os11g03110* | GGGAATCTTGACCCGGAGAA | CTCAGATCCTGCTCCACCAT |
