# Supplementary material for: Mining candidate gene for rice aluminum tolerance through genome wide association study and transcriptomic analysis
Source: BMC Plant Biol. 2019 Nov 12;19:490. doi: 10.1186/s12870-019-2036-z (PMC6852983; doi:10.1186/s12870-019-2036-z)
Supplement: Supplementary file 7 — Additional file 7: Table S3. Summary of categorized SNPs and Indels. [file 12870_2019_2036_MOESM7_ESM.docx]

**Additional file 7: Table S3** Summary of categorized SNPs

| **Location category** | **SNPs** |
| --- | --- |
| promoter | 1,745,893 |
| three_prime_UTR | 223,704 |
| exon | 27,776 |
| CDS | 386,562 |
| five_prime_UTR | 126,732 |
| intron | 1,298,163 |
| Total | 3,808,730 |
